# Protein synthesis and quality control in aging

**DOI:** 10.18632/aging.101721

**Published:** 2018-12-18

**Authors:** Aleksandra S. Anisimova, Alexander I. Alexandrov, Nadezhda E. Makarova, Vadim N. Gladyshev, Sergey E. Dmitriev

**Affiliations:** ^1^Belozersky Institute of Physico-Chemical Biology, Lomonosov Moscow State University, Moscow 119234 Russia; ^2^School of Bioengineering and Bioinformatics, Lomonosov Moscow State University, Moscow 119234 Russia; 3 Bach Institute of Biochemistry of the Russian Academy of Sciences, Moscow 119071 Russia; 4 Division of Genetics, Department of Medicine, Brigham and Women’s Hospital and Harvard Medical School, Boston, MA 02115, USA; 5 Engelhardt Institute of Molecular Biology, Russian Academy of Sciences, Moscow 119991, Russia

**Keywords:** translation, aging, lifespan, proteostasis, ribosome

## Abstract

Aging is characterized by the accumulation of damage and other deleterious changes, leading to the loss of functionality and fitness. Age-related changes occur at most levels of organization of a living organism (molecular, organellar, cellular, tissue and organ). However, protein synthesis is a major biological process, and thus understanding how it changes with age is of paramount importance. Here, we discuss the relationships between lifespan, aging, protein synthesis and translational control, and expand this analysis to the various aspects of proteome behavior in organisms with age. Characterizing the consequences of changes in protein synthesis and translation fidelity, and determining whether altered translation is pathological or adaptive is necessary for understanding the aging process, as well as for developing approaches to target dysfunction in translation as a strategy for extending lifespan.

## Importance of protein synthesis and proteome function in aging

Aging is characterized by the accumulation of various forms of damage as well as by other age-related deleterious changes [1–3]. These changes generally have negative, deleterious consequences for organisms as they age. Different living systems differ in their metabolic strategies, resulting in different types and levels of damage production, therefore have evolved both unique and common mechanisms to counteract some of these deleterious changes. These mechanisms also limit the transfer of damage to progeny. The damage-producing and protective mechanisms are mostly genetically controlled, differ among taxonomic groups and are important in defining the lifespan of organisms. Nevertheless, the general principles of cell and organismal organization make damage accumulation inevitable for most multicellular organisms.

**Figure 1:**
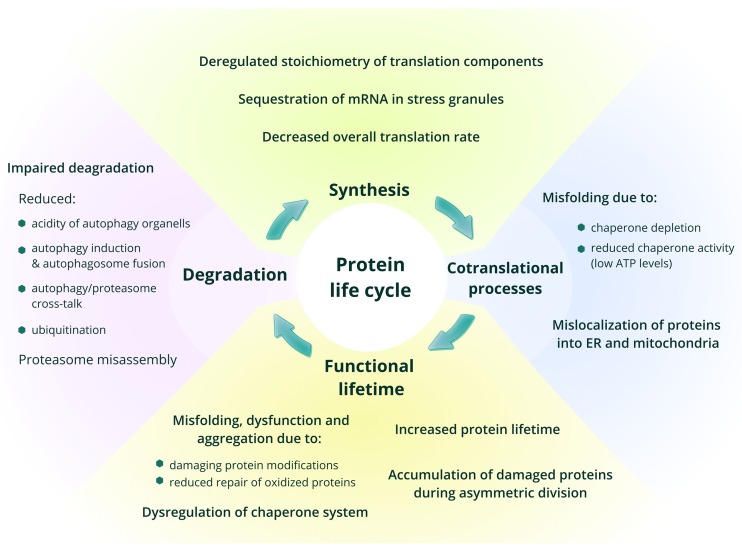
**Age-related changes in protein synthesis and proteostasis.** The main phases in the life of proteins are shown, with a focus on processes that change with age.

In this review, we discuss age-related changes in one of the most important and abundant components of any cell, and therefore of the whole organism – the proteome. Functionality of the whole system of proteins in any organism requires maintenance of a precise balance of synthesis, degradation and function of each and every protein, while aging often shifts this balance, resulting in pathology [4]. Being the end-point of the implementation of genetic information, the proteome accumulates damage generated during this process. The effectiveness of proteostasis control systems, which maintain and recycle the proteome, is diminished with age, leading to the accumulation of damaged proteins and molecules, which in turn inhibit cell functionality and thus cause age-related dysfunction [5]. Every step in protein lifecycle, most notably protein synthesis and degradation, is relevant to the aging process and, indeed, has been shown to change with age and likely define lifespan (Figure 1). While changes in protein degradation systems during aging are relatively well studied, alterations in protein synthesis still remain to be elucidated. Does the overall level of protein synthesis change with age? Which components of the translation apparatus are affected by aging? Do errors in protein synthesis increase in older organisms? Is there age-dependent regulation of protein synthesis at the level of translation? Answering these questions is necessary for understanding the mechanisms of aging and lifespan control. We will focus on them in this review.

## Age-related changes in protein folding and degradation

The life of any protein in the cell begins with synthesis, which is accompanied or followed by co- or post-translational folding and modification, localization to its appropriate compartment, functioning, and, finally, degradation (Figure 1). The apparatus involved in these steps is known as the proteostasis machinery. During or after synthesis, proteins adopt their intended secondary and higher-level structures in a process called folding. In most cases, folding is facilitated by the activity of chaperones and partner proteins. Decreased chaperone capacity with age was shown in numerous studies, and it is clear that it may be affected at multiple levels, including the induction of the chaperone response and chaperone activity (reviewed in [6]). The decision whether to degrade a protein is, among other things, influenced by the availability of ATP in the cell. Deterioration of cellular energetics with age and disruption of fatty acid and glucose metabolism reduce the amount of available ATP, changing chaperone activity and thus leading to the accumulation of damaged proteins [7].

Another facet of proteostasis is the control of protein aggregation, which is also facilitated by chaperones. Increased proteome stability in the form of resistance to aggregation was observed in long-lived compared to closely related shorter-lived groups of bivalve mollusks [8]. Comprehensive proteome profiling revealed imbalance of proteostasis components with age and an overload of chaperone machinery due to increased protein aggregation [9]. Accordingly, upregulation of proteostasis maintaining machinery usually has a positive effect on lifespan. Constitutive activation of the unfolded protein response (UPR) pathway through deletion of some UPR target genes increased yeast replicative lifespan via improved protein homeostasis and induction of various cytoprotective mechanisms [10]. In addition, slow-aging mouse models as well as mice treated with diets and drugs that extend lifespan, were found to have higher levels of ATF4 protein, a major component of the UPR pathway [11,12]. These findings suggest a positive link between moderately elevated stress response and extended lifespan, a paradox consistent with hormesis [13].

Systems maintaining proteostasis are required to repair proteins damaged in the course of their lives [4,5,14]. As an example, methionine oxidation was found to increase with age [15,16], whereas methionine sulfoxide reductases, enzymes responsible for the repair of oxidized methionines in proteins, are depleted during senescence of human fibroblasts, and aging of mice and rats [15,17–20]. Intriguingly, proteins of the naked mole rat, *Heterocephalus glaber*, an organism with the lifespan 10-fold higher than of other rodents of similar body weight, are less susceptible to oxidative damage accumulation [21], despite increased levels of ROS-mediated damage at earlier stages of life of these animals. However, the levels of repair proteins, methionine sulfoxide reductase A and glutaredoxin, are similar between the mouse and the naked mole rat, and the mechanisms that support the increased resistance of the naked mole rat proteome to oxidation remain unknown.

Normal proteins that have reached the end of their lifetime, as well as damaged proteins are degraded by the autophagic and proteasome systems. Autophagy and proteasome-mediated proteolytic activity also deteriorate with age [5,14,22–25]. Their insufficient activity manifests at different levels: the proteasome assembly from its components is imbalanced and accompanied by the alterations in the ubiquitin ligation machinery and an overall reduced level of ubiquitination. In turn, autophagy is impaired both at the stage of induction and fusion of autophagosomes with lysosomes [5,22,26]. Under normal circumstances, these two systems are interconnected and can compensate for the defects in each other’s function. However, aging seems to disrupt the balanced cross-talk between proteostasis modules, so the organism becomes more sensitive to stress [27,28]. Interestingly, age-related impairment of the proteasome systems is less pronounced in long-lived species [21].

Synthesis and degradation of proteins are plastic and mostly tuned to achieve the appropriate protein levels and support necessary changes in response to various stimuli. Proteins are characterized by specific lifetimes, which depend on protein function, location, occurrence in complexes, metabolic status of the cell and other factors. Disruption of a normal life cycle of a protein may affect the lifespan of an organism, as well as the emergence of pathologies. Changes in the turnover rates may also lead to the accumulation and aggregation of proteins, abnormal post-translational modifications and changes of relative concentrations of various proteins, affecting stoichiometry of protein complexes [22]. Indeed, a number of studies have demonstrated the decreased turnover rate and increased lifetime of proteins during aging in various organisms (summarized in [29]). Particular long-lived proteins were shown to be retained in mother cells during yeast replicative aging and accumulated damaging modifications, possibly contributing to the aging process [30]. On the other hand, Yang and co-authors [31] found that proteins with normal life-times that were retained in mother cells during asymmetric division reduced the lifespan of the mother cell. It is important to note that yeast replicative lifespan is limited to several days, whereas many human cells persist for tens of years. Thus, spontaneous amino acid modifications and other forms of protein damage may play a much more important role in the context of human aging.

A recent study of protein turnover in the worm *Caenorhabditis elegans* confirmed the reduced turnover rates for 40% of studied proteins with age. Interestingly, the most prominent decrease was found for ribosomal proteins and proteins participating in translation regulation [32]. On the contrary, studies in mammals did not detect any global changes of protein turnover dynamics during aging or even identified a slight reduction in protein half-lives with age [22,33,34]. Nevertheless, in neurons altered stoichiometry of multiprotein complexes components, such as the nuclear pores, was found to be due to the decreased amounts of long-lived proteins [35]. Changes of turnover rates for certain groups of proteins, such as mitochondrial proteins, were also observed in the muscle [34]. Another well-established example of proteins that experience age-related changes are crystallins, whose damage is associated with cataract, a common age-related pathology [36].

Despite the fact that age-related changes in the turnover rate were not detected in mammals, protein degradation and turnover, apparently, contribute to lifespan control across species. A recent study by the Ghaemmaghami group has shown a negative correlation between the turnover rates and lifespan for eight rodent species [37]. In agreement with previous findings, it supports the beneficial role of decreasing the protein turnover rates in lifespan extension, probably, due to reduced energy consumption. Moreover, the observed impact of turnover on species lifespan seemed to be dependent on differences in protein sequence, but not on the function of protein degradation machinery, therefore making protein sequence another contributor to proteome stability regulation in lifespan control.

Studies of protein metabolism in the context of aging remain rather laborious and have limitations, especially in the case of complex organisms. For instance, these studies often do not account for differences in protein turnover across cellular compartments and tissues. Also, the duration of pulse labeling of a probe may not be sufficient to analyze long-lived proteins, whereas the time between labeling and fixation of tissue may be too long to determine the turnover rate of short-lived proteins [22]. Nevertheless, studies that succeed in identifying alterations in protein turnover with age are consistent in claiming its slow-down. Moreover, in the case of accelerated aging, an increase in turnover rates and protein synthesis was described [38].

## Protein synthesis in aging and lifespan control

### Changes in protein synthesis rate and translation machinery with age

**Figure 2:**
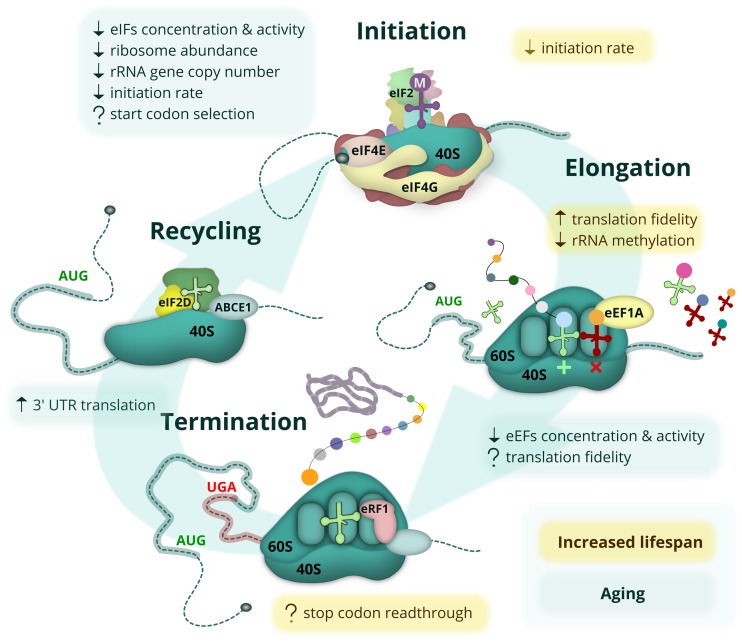
**Age-related changes and lifespan modulating aspects of protein synthesis.** The eukaryotic mRNA translation cycle is shown. During translation initiation, the 43S complex is formed (top). It harbors the initiator Met-tRNAi delivered by eIF2, which is inactivated upon amino acid starvation, UPR or other stress conditions. The 43S complex is loaded onto the capped mRNA 5’ end with the help of eIF4F, composed of the cap-binding protein eIF4E, a scaffold protein eIF4G, and a helicase eIF4A (not shown). The eIF4E-eIF4G interaction is inhibited by 4E-BP repressor proteins, which are activated during amino acid starvation when mTOR kinase is inactive. During elongation (right), cognate (or sometimes near-cognate) aminoacyl-tRNAs are delivered to the translating ribosome by eEF1A, followed by the peptidyl transferase reaction and eEF2-assisted translocation step (not shown). When the ribosome encounters a stop codon, translation termination occurs (bottom). At this step, the synthesized polypeptide is released by termination factor eRF1, delivered by eRF3 (not shown) and assisted by ABCE1. In some cases, however, the stop codon can be recognized by a non-cognate tRNA, leading to a readthrough event. At the final step (left), ribosome and deacylated tRNA should be removed from the mRNA (recycled) with the help of ABCE1, eIF2D and/or MCT-1/DENR proteins. Most of these events are affected by aging (light-green boxes) or linked to lifespan control (yellow boxes). Known positive and negative effects are shown by up and down arrows, respectively, while controversial or potential regulation is indicated by a question mark.

Unlike changes in protein folding, maintenance and degradation, age-related alterations of protein synthesis have not yet been studied in great detail; however, some initial important observations have been made (Figure 2). During the second part of the 20^th^ century, multiple studies demonstrated that the overall level of protein synthesis is reduced with age in various invertebrates, mice, rats and humans, both in different tissues and *in vitro.* These studies observed reduced ribosome abundance, attenuated activity and levels of major initiation and elongation factors (reviewed in [29,39,40]), and also a reduction in the rate of mitochondrial protein synthesis [41] as a function of age. The reduction in protein synthesis is probably a common feature for all living creatures; it has been observed in replicatively aged yeast [42], and, more recently, *in vivo* in sheep using the incorporation of a radioactive amino acid [43].

Several studies also assayed the age-related changes in the expression of genes encoding the components of the translation machinery. Reduction in the level of mRNAs encoding four large ribosome subunit proteins was observed using an RT-qPCR assay of cataract-affected lenses obtained from patients of various ages, but no difference in the mRNA levels of translation factors was found [44]. Nevertheless, at the protein level, reduction of translation elongation factor eEF1A abundance and activity was shown in aging adult *Drosophila melanogaster*, and this decline was suggested to be the main cause of the decreased synthesis of total protein in this case [45], although this conclusion was challenged by the later data [46]. In another study, a two-fold decrease in eEF2 protein abundance was detected in the pineal gland of old rats [47]. Translation elongation factors from young and old rats were also assayed in a cell-free mammalian system and found to be more active in the case of the young eEF1A, but similar for eEF2 preparations [47].

Aging can affect not only the abundance of translation machinery components, but also their ability to control the intracellular distribution of newly synthesized proteins. For example, a decrease in the availability of the nascent polypeptide-associated complex (NAC) due to its partial aggregation during aging may result in mistargeting of co-translationally imported mitochondrial proteins to the endoplasmic reticulum [48,49].

As the rate and type of damage accumulation with age seems to differ for various organs and tissues [34,50–57], changes in protein synthesis are also likely to vary. For example, one study observed an age-related reduction in total mRNA, as well as in the levels of initiation and elongation factors and RNA polymerase I protein in rat fast plantaris muscle, but not the slow soleus muscle [58]. It was also reported that the brain shows altered translation efficiencies for 15% of analyzed transcripts, compared to 2% in the liver [59].

Interestingly, the data on changes in expression of genes encoding ribosomal proteins in replicatively aged yeast are somewhat contradictory. On the one hand, increased amounts of ribosomal proteins were observed using mass-spectrometry-based proteomics [60], but on the other, the output of ribosomal protein mRNA translation as well as the overall translation efficiency identified with ribosome profiling were shown to decline with age [61]. This may indicate some defects in the degradation of ribosomes. The alterations of translation with the increasing replicative age of yeast were also confirmed by the observed gradual uncoupling of protein levels from the levels of their transcripts [60]. Additionally, several studies reported the disruption of the stoichiometry of translation machinery components with age [9,32,60]. Notably, the translational output, measured by ribosome profiling in the brains of young and old rats, was increased for ribosomal proteins, but decreased for translation factors, providing another example of translation machinery deregulation with age [59].

The abundance of ribosomes may even affect lifespan within species. The distribution of ribosomal RNA gene copy number was shown to be narrowed in the genomes of elderly humans, indicating that there may be an optimal level of ribosomal RNA required. It was hypothesized that a low copy number is not sufficient for maintaining the function of an aging organism, whereas a high copy number may also represent a disadvantage during aging, or, alternatively, the number of gene copy just decreases with age [62]. Somewhat similar results were obtained for *Saccharomyces cerevisiae*, where older cells with the reduced amount of ribosomal proteins exhibited a longer replicative lifespan, while in younger cells, the ribosomal protein abundance correlated positively with lifespan [63].

## Changes of translation fidelity with age

Another important aspect of protein synthesis, which attracts the attention of researchers in the aging field, is the occurrence of translational errors, namely decoding errors and stop codon readthrough events. Attempts to assay age-related changes in translational fidelity have been made since the 1970s. The initial reports on this line of enquiry were rather controversial, unlike the data on protein synthesis and degradation. Many of them used cell-free translation systems or ribosomes isolated from organs of animals of different ages [64–66]. The authors of these reports were unable to detect age-related changes in translation fidelity.

Translational fidelity was also studied in extracts obtained from aging cultures of primary fibroblasts. The results of these studies were also controversial. For instance, one study noted that the number of translational errors in an extract obtained from fibroblasts that had completed around 55 doublings was 7-fold higher than that of cells which had doubled 28 times [67]. On the other hand, no change in translational fidelity was found in a study which investigated human skin fibroblasts obtained from healthy subjects and progeria patients and compared the fidelity of translation in cells from early and late passages [68]. Another study identified differences between ribosomes isolated from young and old animals. The authors used paromomycin, an aminoglycoside antibiotic, which decreases translational accuracy. Ribosomes isolated from the livers of old rats exhibited increased sensitivity to this antibiotic, i.e. after treatment with paromomycin they made 9% more errors than the ribosomes obtained from young rats [66].

The contradictory results of these studies may be due to the use of cell-free translation systems to assess translational fidelity [69]. For instance, the frequency of translation errors (misincorporation of amino acids) in yeast was estimated to be 10^-5^ [70]. However, estimates of fidelity from one of the most widely used systems for *in vitro* translation, rabbit reticulocyte lysate, are highly variable - from 10^-5^ [71] to 10^-4^ [72]. Such a discrepancy precludes reliable estimation of changes in translational fidelity with age. Another drawback of these early studies was the use of very specific systems to assess accuracy of amino acid incorporation, which were chosen due to the absence of more appropriate methods at the time. For instance, Luce and Bunn [67] used a purified mRNA encoding the coat protein of the cowpea variant of tobacco mosaic virus. This protein lacks any cysteine residues, and errors were detected using an aberrant incorporation of the labeled cysteine, while another study [65] assayed incorporation of leucine during the translation of a synthetic poly(U), which normally encodes poly-phenylalanine. Such systems do not recapitulate the translation of real cellular mRNAs, and the obtained estimates do not include errors in tRNA aminoacylation. Moreover, the protocol for preparing the cell-free *in vitro* translation system may introduce major changes in the parameters of protein synthesis. Thus, we should bear in mind that the shortcomings of cell-free *in vitro* systems may mask differences between studied samples. For these reasons, more recent studies aiming to assay the relation between translational fidelity and aging were performed in living cells. One study addressed changes in translation fidelity of replicatively aged yeast upon transformation of live cells with luciferase reporters [73]. This approach did not detect changes in translation fidelity.

The impairment of ribosome recycling was recently described in the aging mouse brains [74]. It was shown that the brain regions that are particularly sensitive to oxidative stress are enriched in short RNAs representing isolated 3’ untranslated regions (UTR) of the regular mRNAs. These fragments are, apparently, the mRNA decay intermediates accumulated due to stalling of unrecycled ribosomes at the 3’ UTRs under conditions when the ribosome recycling factor ABCE1 is damaged by oxidative stress. Translation of these RNAs results in the production of short peptides. Although the accumulation of mRNA fragments and peptides in the aging cell has not yet been shown to be a damaging factor, it likely reflects impairment of protein synthesis fidelity and could serve as a biomarker of aging.

## The number of translation errors is correlated with lifespan

Despite contradictory results on translational fidelity in aging organisms, nature has left some important clues with regard to relevance of translation fidelity to organismal aging, which was revealed by several studies that analyzed this process in various animal species [75,76]. The naked mole rat, a rodent with an exceptionally long lifespan for its body mass, is thought to have translation fidelity nearly 10-fold higher than that of the mouse [75]. This study used fibroblasts isolated from the skin of mice and naked mole rats and transfected with various luciferase-based reporter constructs. They encoded firefly luciferase, but had either point mutations in the sequence, thus changing key amino acid residues, frameshifts, or premature stop-codons. Luminescence of luciferase in the cells transfected with these constructs could thus only be detected due to translation errors. The study used six constructs for assaying the following errors: three constructs for each codon position, one for readthrough and two for +1 and -1 nucleotide frameshifts. Notably, the authors used DNA-based constructs for transfection, thus the signal should be sensitive to both translation and transcription errors; however, the latter seemed to be negligible, as a clear dependence of the error rate on nucleotide position within the codon was observed. In addition to translation accuracy, the overall protein synthesis of the mouse and naked mole rat was also measured, but no difference was observed [75].

Using the same panel of constructs, translation fidelity was assayed in cultured primary fibroblasts of 17 rodents with different lifespan (4 to 32 years) and body weight [76]. The results showed that the level of error in the first and second, but not the third codon position is negatively correlated with the maximal lifespan, but not with the body weight of 16 out of the 17 species. It is possible that errors in the third (wobble) codon position are less critical, more loosely controlled and do not affect the evolution of lifespan, or that the errors are mainly due to the aminoacyl-tRNA-synthases, and not due to decoding errors by the ribosome. The rate of stop codon readthrough did not correlate with the lifespan too, suggesting a higher selective pressure for translation termination fidelity due to the high energetic cost of failing to recognize a stop codon [76].

In yeast, translational errors caused by antibiotics were shown to impair the folding of proteins and activate the same chaperone groups, which are activated during aging, such as Hsp104 and Hsp26 [73], and translation accuracy impairment induced by several methods decreased the chronological lifespan. The authors hypothesized that the increase in translational errors coupled with the increase in misfolded protein levels can overload the chaperone system, thus explaining the effect of translation errors on lifespan [73]. Notably, mistranslation due to the defective aminoacyl-tRNA has also been reported to be mutagenic, thus aggravating its impact in the deleteriome [77]. In the filamentous fungus *Podospora anserina*, strains bearing high fidelity mutations in the eEF1A gene were shown to have drastically increased longevity [78].

To sum up, as of now, it seems that aging is not accompanied by dramatic decreases in translational fidelity. But the large body of data that demonstrates no significant change with age is counterbalanced by indirect indications of the possible relationship, such as the sensitivity of ribosomes isolated from old animals to antibiotics, or the positive correlation between translation fidelity and maximal lifespan. As proposed by von der Haar and colleagues [73], it is possible that modification of translation fidelity is a factor that controls lifespan between different groups of organisms, but it seems not to play a role in lifespan plasticity of single organisms. The situation resembles that of protein turnover rate contribution to aging in mammals (see above), which is not shown to be directly altered during aging, but is negatively correlated with lifespan of rodent species [37].

## Extending lifespan through manipulation of protein synthesis and translation-related signaling

### Manipulation of translational machinery can modulate lifespan

An aging organism experiences an overall reduction of protein synthesis rates (see above). However, there is another line of evidence that highlights the importance of translation changes in aging and their effects on health. Namely, there are numerous examples of interventions affecting protein synthesis, which modulate lifespan. Sometimes, such modifications are very peculiar, as in the case of ribosomal RNA methylation at a single residue that was shown to be implicated in the control of lifespan in flies, worms and yeast [79]. But a notable and well established phenomenon, connecting translation with aging is that decreasing overall translation by various means can increase lifespan (reviewed in [39,49,80,81]). For example, this can be done by affecting translation initiation. Deletion or downregulation of the IFE-2 somatic isoform of the initiation factor eIF4E of the worm *C. elegans* enhances the effects of pro-longevity mutations *age*, *daf, clk* and *eat* [82]. Downregulation of another initiation factor, eIF4G (IFG-1) increases the average lifespan of nematodes by more than 30% [83,84], while deletion of two subunits of eIF3 results in a 40% extension [85]. The nematode lifespan is also improved by downregulation of one of the subunits of the eIF2B initiation factor [86]. A search for pro-longevity genes using RNA-interference in *C. elegans* identified ribosomal proteins and numerous components of the ribosome biosynthesis apparatus, as well as initiation factors eIF1, eIF4E, eIF4G, eIF4A, eIF2B, eIF5A and subunits of factors eIF2 и eIF3 [87–91]. Decreased translation level in flies achieved by acute overexpression of the regulatory factor 4E-BP lowered protein aggregation in the muscle and extended lifespan [92,93]. Similar observations were also made in the budding yeast and filamentous fungi: a reduction in the levels of several ribosomal proteins [94] or factors involved in the biogenesis of the 60S ribosomal subunit [95] as well as mutations in translation factors [96] resulted in increased replicative lifespan of asymmetrically dividing *S. cerevisiae*, while mutations in some ribosomal proteins also extended lifespan in *Podospora anserina* [97]. Somewhat controversial results were obtained for translation elongation factors. In early studies, fruit flies with elevated expression of eEF1A were shown to live longer, while later experiments revealed no effects on lifespan (summarized in [98,99]). For elongation factor eEF2, inhibition by EFK-1 kinase was shown to be important for the longevity of *C. elegans* both during starvation and under nutrient-rich conditions [100], confirming an overall positive effect of a decreased translation rate on longevity.

One of the ways in which reduced protein synthesis is thought to prolong life is the reduction of the load borne by the chaperone machinery [101]. Translation attenuation by the antibiotic minocycline enhances longevity and proteostasis in *C. elegans* by lowering the concentration of newly synthesized aggregation-prone proteins [102]. Since protein synthesis is the most energy-consuming process in the cell [103], its reduction could also lead to reallocation of energy to processes that repair and compensate various types of damage in the cell, as well as those that reduce damage generated in this process. Additionally, the slow-down of growth rates and consequently of the rates of protein synthesis increases the accuracy of translation, and therefore the proteome quality and stability [73].

Strikingly, together with the decrease in actively translating ribosomes, dietary restriction in mice decreased ribosome lifetime by almost 15% and increased rRNA and ribosomal protein turnover rates [104]. It appears that renewal of the intracellular ribosome pool leads to a lower level of translational errors thus improving proteostasis. In agreement with the effects of dietary restriction, rapamycin-induced inhibition of mTOR, one of the major hubs of nutrient signaling (see below), also increased ribosome turnover rates [105].

Mitochondrial translation contributes to longevity and lifespan control as well. In replicatively aging yeast, non-specific inhibition of mitochondrial translation [106], as well as deletion of mitoribosomal protein Afo1 [107] and the Afg3 protease involved in mitoribosome maturation [108] can increase replicative lifespan. It is likely that these interventions act through their effects on cytoplasmic translation and the integrated stress response pathway [109].

### Signaling pathways controlling longevity via modulation of protein synthesis

**Figure 3:**
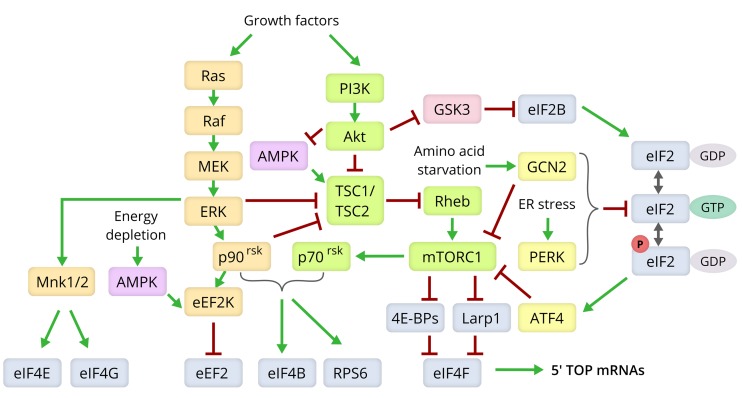
**Molecular pathways modulating lifespan via control of the translation machinery**. Components of the Ras/MEK/ERK signaling pathway are shown in yellow, those of the PI3K/Akt/mTOR axis in green, the ISR pathway in yellow, and the translation machinery components in grey [80,166,175,180].

Most of the established methods for increasing lifespan are based on slowing down the metabolism using dietary interventions or by affecting the molecular cascades controlling metabolism [80,110]. These cascades stimulate overall protein synthesis, are in tight coordination with each other, and also dampen protein degradation [111]. Figure 3 depicts the molecular pathways that link the various strategies of lifespan extension with the components of the translation machinery.

Compounds that robustly expand the lifespan of many model organisms, rapamycin and its analogs (“rapalogs”) [112–115], affect translation via the mTORC1 signaling pathway [116,117]. mTORC1 and mTORC2 are multiprotein kinase complexes with a shared catalytic subunit, mTOR [118]. The precise mechanism by which how mTORC1 affects the synthesis of translation machinery components is still unclear [119,120], although its signaling connections, putting it in the center of the metabolic control of cell growth, have been studied extensively [111,121].

The two main mTORC1 targets that affect translation are 4E-BP and ribosomal protein S6 kinase (p70^rsk^) [111,122]. mTOR phosphorylates the cap-dependent translation regulators of the 4E-BP family, thus inactivating them. Direct mTOR kinase inhibitors, such as Torin1 or PP242, cause dephosphorylation of 4E-BPs and decrease translation efficiency, since the hypophosphorylated forms of 4E-BPs bind the cap-binding protein eIF4E, preventing its interaction with its partner eIF4G and other components of the translation apparatus [111,119]. Although rapamycin is also usually considered as an mTOR inhibitor, it acts allosterically and binds to the kinase indirectly through immunophilin FKBP12, exclusively in the context of the mTORC1 complex [111]. Accordingly, it has different effects on mTOR targets (e.g. 4E-BP1) and overall mRNA translation than the direct (ATP-competitive) mTOR inhibitors [123–127] and extends lifespan even in 4E-BP-null organisms [93].

Another major mTORC1 target is S6 kinase which, among others, phosphorylates the S6 protein of the 40S ribosomal subunit and the eIF4B initiation factor (cofactor of the eIF4A helicase) [123,124,128,129]. Relative impact of S6K and mTOR kinases on translation activation and their cross-talk in the response to extracellular stimuli are still a matter of debate (see [111,127,130] and references therein). However, it is generally accepted that signaling via mTORC1 and S6 kinase axes upregulates translation, while both rapamycin and direct mTOR inhibitors act in the opposite direction [131].

Changes in the mTOR pathway activity during aging is currently an ongoing research direction [132]. Deletion of the TOR1 gene increases replicative lifespan in yeast [94,96]. mTOR downregulation also extends lifespan in worms [133] and flies [134]. Finally, female knockout mice heterozygous for both mTOR and another mTORC1 component, mLST8, also live longer [135]. However, no common trend in mTOR pathway activity changes with age could be detected in mice, as it is easily influenced by numerous environmental and intrinsic conditions (e.g. sex, tissue, feeding status) [136]. Nevertheless, translation downregulation caused by artificially elevated activity of 4E-BP improves proteostasis and extends lifespan in flies similarly to mTORC1 inhibition [92,93,137]. The p70^rsk^ signaling, which is connected to mTORC1, was shown to be elevated in old mice [33], and the animals lacking S6K1 or overexpressing dominant-negative forms of mTOR or S6K exhibited extended lifespan [134,138].

Most components of the translation apparatus (ribosomal proteins, elongation factors, some RNA-binding proteins and translation initiation factors) contain a 5' terminal oligopyrimidine tract (5' TOP) at the beginning of their mRNAs [120,139]. This specific sequence usually starts with a cytosine followed by 4 to 15 pyrimidine bases, and then by a GC-rich region. The 5' TOP motif is highly conserved among mammals and is also found in the mRNAs of some ribosomal proteins of fruit flies; however, it has not been found in lower eukaryotes [120]. It is thought that the 5' TOP sequence mediates the effect of mTORC1 on the translation of mRNAs encoding proteins of the translation apparatus [120,122]. The current consensus on the mechanism of action of rapamycin and direct inhibitors of mTOR on cellular translation is as follows: these compounds cause a subset of mRNAs (mostly those containing 5' TOP), which comprise up to 80% of overall translation in actively proliferating cells, to be selectively liberated from polysomes [125,140]. One possible explanation of this phenomenon is based on the different affinity of the cap-binding protein eIF4F to the 5' UTRs of various mRNA, as well as their competition with other mRNA-binding proteins, including those with the high affinity to the 5' TOP motif [141]. TIA-1/TIAR and/or LARP1 may be the proteins that accomplish regulation of these processes in various physiologic conditions. It has been shown that TIA-1/TIAR suppress the translation of 5' TOP mRNAs and promote their localization to stress granules during amino acid starvation [142]. Nevertheless, they do not mediate repression of 5' TOP mRNA in conditions of insufficient serum or oxygen [143]. The data on the other factor, LARP1, are somewhat contradictory. On the one hand, it has been shown that LARP1 is associated with the mTORC1 complex and actively translating mRNAs, while downregulation of LARP1 causes selective suppression of 5' TOP mRNA translation [144]. On the other hand, LARP1 directly binds the mRNA cap, competing with the assembly of eIF4F and translation initiation [145]. Moreover, depletion of LARP1 increases translation of 5' TOP mRNAs and makes them resistant to inhibition by rapamycin [146,147]. Thus, a two-step model of 5' TOP mRNA regulation via mTORC1 is plausible. Inhibition of mTORC1 causes suppression of translation via activation of 4E-BPs and inactivation of S6K; however, different mRNAs react quite differently to this inhibition. 5' TOP mRNA are much more sensitive to suppression, since LARP1 probably overcompetes eIF4F in binding to the 5' TOP mRNA’s termini under these conditions due to the decreased affinity of these regions to eIF4E and/or eIF4G, eIF4F components [141,148,149].

5' TOP is not the only structural feature dictating the specific regulation of mRNAs encoding translation components. Recently, RNA-binding protein HuD was shown to regulate many mRNAs encoding mTORC1-responsive ribosomal proteins and translation factors via regulatory elements in their 3' UTRs [150]. Intriguingly, mTORC1 activation also causes mRNA 3' UTR shortening in a transcriptome-wide scale, leading to enhanced polysome recruitment of 3' UTR-shortened transcripts [151]. Contrary to this, a widespread 3' UTR lengthening of mRNAs has recently been discovered during cellular senescence [152]. This implies a cross-talk between mTOR-dependent regulation at transcriptional and translational levels under normal, stress and pathological conditions.

It should be noted that the mTORC1/eIF4F-mediated translational control is widely known, but it is not the only pathway regulating cellular mRNA translation. Although the highly mTOR-dependent mRNAs constitute a majority of transcripts in polysomes of actively proliferating cells, they represent just a minority of mRNA species [125,140]. Many other mRNA species tolerate 4E-BP1 activation. They are likely to be less dependent on eIF4F and even on the 5’ terminal m^7^G-cap, although still require a free 5’ end to recruit the ribosome [141]. To explain efficient mRNA translation under these conditions, a number of alternative molecular mechanisms of ribosome recruitment were recently proposed. These include m^6^A-dependent pathways, mediated by eIF3, ABCF1 and/or YTHDF1/3 [153–156]; special sequences within mRNA leaders [157]; involvement of distinct cap-binding proteins like eIF3d [158], CBC (nuclear cap-binding complex) [159,160] or unconventionally composed eIF4F variants [161,162]; alternative RNA helicases like DDX3/Ded1p [160,162,163] or other non-canonical translation initiation mechanisms. However, their importance to aging and lifespan control are yet to be investigated, although some indirect evidence points to their putative relevance [84,164].

Translation is also regulated through cytokine and hormone induced signaling. PI3K/AKT and MAPK pathways are activated in the presence of insulin and growth factors (Figure 3). They are interconnected with the mTOR pathway and can also regulate translation in an age-dependent manner. Knockout of various components of the PI3K/Akt signaling pathway, like the insulin receptor (*daf*) genes, increases lifespan (reviewed in [165]). Signals from the insulin and growth factor receptors converge on a few signaling hubs, including the Akt kinase, which in turn activate mTORC1, while the kinases ERK1 and ERK2 of the MAPK-pathway activate the S6 kinase (p90^rsk^) directly [111]. ERK1 and ERK2 also activate the Mnk kinase, a positive regulator of translation initiation via phosphorylation of eIF4E and stabilization of its interaction with eIF4G and the 5′ cap structure. The Mnk1 kinase can be also activated by the p38 stress kinase, which is a component of the MAPK-pathway [39,166]. Phosphorylation levels of most of the signaling molecules of this axis (ERK 1/2, p90^rsk,^ Mnk1 and p38 MAPK) were shown to be higher in muscles of older men [167].

Another molecular mechanism, which connects lifespan control and protein synthesis, is the integrated stress response (ISR). One of its branches is initiated by specific translational upregulation of upstream ORF-containing mRNAs encoding stress transcription factors (GCN4 in yeast and ATF4 in mammals). Lowered amino acid levels and appearance of free deacylated tRNAs in the cytoplasm activate the evolutionary conserved GCN2 kinase, which inhibits the eIF2 initiation factor by phosphorylating its α-subunit. Intriguingly, limitation for different amino acids triggers distinct signaling branches and has different effects on translation [168–170], thus providing a molecular basis for a variety of dietary restriction interventions with different potential to extend lifespan. In mammalian cells, three additional kinases exist that phosphorylate eIF2α under various conditions, including PERK, which is activated upon endoplasmic reticulum stress and triggers the unfolded protein response (UPR). When active eIF2α is lacking, synthesis of most proteins is suppressed; however, a peculiar arrangement of uORFs in the 5′ UTRs of the yeast GCN4 and mammalian ATF4 mRNAs, as well as of some other transcripts, allows their translational induction under these conditions [171,172]. The transcription factors induce the expression of genes necessary for cell survival. During the ISR, mTOR kinase activity is also repressed, since a cross-talk exists between the two signaling pathways (see, for example [173,174]). Ribosome biogenesis usually decreases while autophagy is increased during ISR, assisting proteostasis maintenance under stress conditions. Therefore, the ISR improves protein folding and maintains proteostasis [175], with an emerging role of mTORC1 in this process [176].

The role of ISR in lifespan extension is relatively well studied in yeast. GCN4 was shown to be at least partially involved in both replicative and chronological lifespan extension by various ways including depletion of nutrients and ribosomal components (reviewed in [109]). Elevating the ISR level is observed during aging in different species, likely because of decreased protein quality with age together with the depletion of energy resources. In replicatively aged yeast, protein synthesis was reduced due to activation of stress-induced Gcn2 kinase and by mRNA re-localization to P-bodies mediated by the Ssd1 mRNA binding protein [61], whereas in aged rats increased expression of ATF4 was observed in the retina, also possibly indicating induction of a protective program in response to impaired proteostasis [177].

Translation elongation rate is also regulated by intra- and extracellular stimuli and conditions, including starvation. AMPK, which is activated during energy depletion, phosphorylates eEF2K, a specific kinase for translation elongation factor eEF2 (Figure 3). This phosphorylation leads to a decreased eEF2 translocase activity and slows down elongating ribosome velocity [111]. *C. elegans* deficient in EFK-1, an ortholog of eEF2K, had a shorter lifespan not only in response to starvation, but also under nutrient-rich conditions [100], while the increase in AMPK subunit levels, in turn, prolonged the worm’s life [178].

## CONCLUSIONS

Deregulation of nutrient signaling and accumulation of damage in the aging proteome lead to decreased protein synthesis. This decrease seems to serve as an adaptation of the organism to age-related changes and may be beneficial in terms of longevity, as downregulation of protein synthesis and an increase in proteome stability are associated with increased lifespan. The effects of aging on the proteome, and more specifically, on the dynamics of protein synthesis are, as of yet, incompletely understood, and elucidation of how lifespan is controlled in various organisms and what factors shape the aging trajectory of a specific organism or its different tissues is an important goal for future studies. Currently, only a few reports have performed omics-level analyses of protein synthesis in the context of aging and nearly all of them used only two age groups (younger and older), whereas many other studies addressed changes in either highly specialized tissues, or did not compare tissues with one another. However, data on the rates of damage accumulation in different tissues [50–57] as well non-linear changes in transcriptomic readouts during aging [179] indicate that studies with more temporal resolution, i.e. more age points, involving different tissues are needed to gain a better and deeper understanding of age-related changes. In turn, these studies may help identify the important drivers of deterioration in specific tissues and pave the way for developing effective longevity interventions.

## References

[1] Zimniak P. Detoxification reactions: relevance to aging. Ageing Res Rev. 2008; 7:281–300. 10.1016/j.arr.2008.04.001 18547875 PMC2671233

[2] Gladyshev VN. On the cause of aging and control of lifespan: heterogeneity leads to inevitable damage accumulation, causing aging; control of damage composition and rate of accumulation define lifespan. BioEssays. 2012; 34:925–29. 10.1002/bies.201200092 22915358 PMC3804916

[3] Gladyshev VN. Aging: progressive decline in fitness due to the rising deleteriome adjusted by genetic, environmental, and stochastic processes. Aging Cell. 2016; 15:594–602. 10.1111/acel.12480 27060562 PMC4933668

[4] Taylor RC, Dillin A. Aging as an event of proteostasis collapse. Cold Spring Harb Perspect Biol. 2011; 3:3. 10.1101/cshperspect.a004440 21441594 PMC3101847

[5] Kaushik S, Cuervo AM. Proteostasis and aging. Nat Med. 2015; 21:1406–15. 10.1038/nm.4001 26646497

[6] Soti C, Csermely P. Aging and molecular chaperones. Exp Gerontol. 2003; 38:1037–40. 10.1016/S0531-5565(03)00185-2 14580856

[7] Calderwood SK, Murshid A, Prince T. The shock of aging: molecular chaperones and the heat shock response in longevity and aging--a mini-review. Gerontology. 2009; 55:550–58. 10.1159/000225957 19546513 PMC2754743

[8] Treaster SB, Ridgway ID, Richardson CA, Gaspar MB, Chaudhuri AR, Austad SN. Superior proteome stability in the longest lived animal. Age (Dordr). 2014; 36:9597. 10.1007/s11357-013-9597-9 24254744 PMC4082568

[9] Walther DM, Kasturi P, Zheng M, Pinkert S, Vecchi G, Ciryam P, Morimoto RI, Dobson CM, Vendruscolo M, Mann M, Hartl FU. Widespread Proteome Remodeling and Aggregation in Aging C. elegans. Cell. 2015; 161:919–32. 10.1016/j.cell.2015.03.032 25957690 PMC4643853

[10] Labunskyy VM, Gerashchenko MV, Delaney JR, Kaya A, Kennedy BK, Kaeberlein M, Gladyshev VN. Lifespan extension conferred by endoplasmic reticulum secretory pathway deficiency requires induction of the unfolded protein response. PLoS Genet. 2014; 10:e1004019. 10.1371/journal.pgen.1004019 24391512 PMC3879150

[11] Li W, Miller RA. Elevated ATF4 function in fibroblasts and liver of slow-aging mutant mice. J Gerontol A Biol Sci Med Sci. 2015; 70:263–72. 10.1093/gerona/glu040 24691093 PMC4351389

[12] Li W, Li X, Miller RA. ATF4 activity: a common feature shared by many kinds of slow-aging mice. Aging Cell. 2014; 13:1012–18. 10.1111/acel.12264 25156122 PMC4326926

[13] Shore DE, Ruvkun G. A cytoprotective perspective on longevity regulation. Trends Cell Biol. 2013; 23:409–20. 10.1016/j.tcb.2013.04.007 23726168 PMC4057428

[14] Labbadia J, Morimoto RI. The biology of proteostasis in aging and disease. Annu Rev Biochem. 2015; 84:435–64. 10.1146/annurev-biochem-060614-033955 25784053 PMC4539002

[15] Höhn A, König J, Grune T. Protein oxidation in aging and the removal of oxidized proteins. J Proteomics. 2013; 92:132–59. 10.1016/j.jprot.2013.01.004 23333925

[16] Stadtman ER, Van Remmen H, Richardson A, Wehr NB, Levine RL. Methionine oxidation and aging. Biochim Biophys Acta. 2005; 1703:135–40. 10.1016/j.bbapap.2004.08.010 15680221

[17] Ahmed EK, Rogowska-Wrzesinska A, Roepstorff P, Bulteau AL, Friguet B. Protein modification and replicative senescence of WI-38 human embryonic fibroblasts. Aging Cell. 2010; 9:252–72. 10.1111/j.1474-9726.2010.00555.x 20102351

[18] Novoselov SV, Kim HY, Hua D, Lee BC, Astle CM, Harrison DE, Friguet B, Moustafa ME, Carlson BA, Hatfield DL, Gladyshev VN. Regulation of selenoproteins and methionine sulfoxide reductases A and B1 by age, calorie restriction, and dietary selenium in mice. Antioxid Redox Signal. 2010; 12:829–38. 10.1089/ars.2009.2895 19769460 PMC2864656

[19] Petropoulos I, Mary J, Perichon M, Friguet B. Rat peptide methionine sulphoxide reductase: cloning of the cDNA, and down-regulation of gene expression and enzyme activity during aging. Biochem J. 2001; 355:819–25. 10.1042/bj3550819 11311146 PMC1221799

[20] Vanhooren V, Navarrete Santos A, Voutetakis K, Petropoulos I, Libert C, Simm A, Gonos ES, Friguet B. Protein modification and maintenance systems as biomarkers of ageing. Mech Ageing Dev. 2015; 151:71–84. 10.1016/j.mad.2015.03.009 25846863

[21] Pérez VI, Buffenstein R, Masamsetti V, Leonard S, Salmon AB, Mele J, Andziak B, Yang T, Edrey Y, Friguet B, Ward W, Richardson A, Chaudhuri A. Protein stability and resistance to oxidative stress are determinants of longevity in the longest-living rodent, the naked mole-rat. Proc Natl Acad Sci USA. 2009; 106:3059–64. 10.1073/pnas.0809620106 19223593 PMC2651236

[22] Basisty N, Meyer JG, Schilling B. Protein Turnover in Aging and Longevity. Proteomics. 2018; 18:e1700108. 10.1002/pmic.201700108 29453826 PMC6022828

[23] López-Otín C, Blasco MA, Partridge L, Serrano M, Kroemer G. The hallmarks of aging. Cell. 2013; 153:1194–217. 10.1016/j.cell.2013.05.039 23746838 PMC3836174

[24] Proteasomal DI, Systems AD. Annu Rev Biochem. 2017; 86:193–224. 10.1146/annurev-biochem-061516-044908 28460188

[25] David DC. Aging and the aggregating proteome. Front Genet. 2012; 3:247. 10.3389/fgene.2012.00247 23181070 PMC3501694

[26] Vilchez D, Saez I, Dillin A. The role of protein clearance mechanisms in organismal ageing and age-related diseases. Nat Commun. 2014; 5:5659. 10.1038/ncomms6659 25482515

[27] Schneider JL, Villarroya J, Diaz-Carretero A, Patel B, Urbanska AM, Thi MM, Villarroya F, Santambrogio L, Cuervo AM. Loss of hepatic chaperone-mediated autophagy accelerates proteostasis failure in aging. Aging Cell. 2015; 14:249–64. 10.1111/acel.12310 25620427 PMC4364837

[28] Gavilán E, Pintado C, Gavilan MP, Daza P, Sánchez-Aguayo I, Castaño A, Ruano D. Age-related dysfunctions of the autophagy lysosomal pathway in hippocampal pyramidal neurons under proteasome stress. Neurobiol Aging. 2015; 36:1953–63. 10.1016/j.neurobiolaging.2015.02.025 25817083

[29] Van Remmen H, Ward WF, Sabia RV, Richardson A. Gene Expression and Protein Degradation. Handbook of Physiology, Aging. Hoboken, NJ, USA: John Wiley & Sons, Inc. 1995. 171–234

[30] Thayer NH, Leverich CK, Fitzgibbon MP, Nelson ZW, Henderson KA, Gafken PR, Hsu JJ, Gottschling DE. Identification of long-lived proteins retained in cells undergoing repeated asymmetric divisions. Proc Natl Acad Sci USA. 2014; 111:14019–26. 10.1073/pnas.1416079111 25228775 PMC4191815

[31] Yang J, McCormick MA, Zheng J, Xie Z, Tsuchiya M, Tsuchiyama S, El-Samad H, Ouyang Q, Kaeberlein M, Kennedy BK, Li H. Systematic analysis of asymmetric partitioning of yeast proteome between mother and daughter cells reveals “aging factors” and mechanism of lifespan asymmetry. Proc Natl Acad Sci USA. 2015; 112:11977–82. 10.1073/pnas.1506054112 26351681 PMC4586869

[32] Dhondt I, Petyuk VA, Bauer S, Brewer HM, Smith RD, Depuydt G, Braeckman BP. Changes of Protein Turnover in Aging *Caenorhabditis elegans.* Mol Cell Proteomics. 2017; 16:1621–33. 10.1074/mcp.RA117.000049 28679685 PMC5587862

[33] Basisty N, Dai DF, Gagnidze A, Gitari L, Fredrickson J, Maina Y, Beyer RP, Emond MJ, Hsieh EJ, MacCoss MJ, Martin GM, Rabinovitch PS. Mitochondrial-targeted catalase is good for the old mouse proteome, but not for the young: ‘reverse’ antagonistic pleiotropy? Aging Cell. 2016; 15:634–45. 10.1111/acel.12472 27061426 PMC4933659

[34] Kruse SE, Karunadharma PP, Basisty N, Johnson R, Beyer RP, MacCoss MJ, Rabinovitch PS, Marcinek DJ. Age modifies respiratory complex I and protein homeostasis in a muscle type-specific manner. Aging Cell. 2016; 15:89–99. 10.1111/acel.12412 26498839 PMC4717270

[35] Toyama BH, Savas JN, Park SK, Harris MS, Ingolia NT, Yates JR 3rd, Hetzer MW. Identification of long-lived proteins reveals exceptional stability of essential cellular structures. Cell. 2013; 154:971–82. 10.1016/j.cell.2013.07.037 23993091 PMC3788602

[36] Pescosolido N, Barbato A, Giannotti R, Komaiha C, Lenarduzzi F. Age-related changes in the kinetics of human lenses: prevention of the cataract. Int J Ophthalmol. 2016; 9:1506–17. 10.18240/ijo.2016.10.23 27803872 PMC5075670

[37] Swovick K, Welle KA, Hryhorenko JR, Seluanov A, Gorbunova V, Ghaemmaghami S. Cross-species Comparison of Proteome Turnover Kinetics. Mol Cell Proteomics. 2018; 17:580–91. 10.1074/mcp.RA117.000574 29321186 PMC5880112

[38] Buchwalter A, Hetzer MW. Nucleolar expansion and elevated protein translation in premature aging. Nat Commun. 2017; 8:328. 10.1038/s41467-017-00322-z 28855503 PMC5577202

[39] Gonskikh Y, Polacek N. Alterations of the translation apparatus during aging and stress response. Mech Ageing Dev. 2017; 168:30–36. 10.1016/j.mad.2017.04.003 28414025

[40] Rattan SI. Synthesis, modifications, and turnover of proteins during aging. Exp Gerontol. 1996; 31:33–47. 10.1016/0531-5565(95)02022-5 8706803

[41] Rooyackers OE, Adey DB, Ades PA, Nair KS. Effect of age on in vivo rates of mitochondrial protein synthesis in human skeletal muscle. Proc Natl Acad Sci USA. 1996; 93:15364–69. 10.1073/pnas.93.26.15364 8986817 PMC26410

[42] Motizuki M, Tsurugi K. The effect of aging on protein synthesis in the yeast Saccharomyces cerevisiae. Mech Ageing Dev. 1992; 64:235–45. 10.1016/0047-6374(92)90081-N 1405782

[43] Connors MT, Poppi DP, Cant JP. Protein elongation rates in tissues of growing and adult sheep. J Anim Sci. 2008; 86:2288–95. 10.2527/jas.2007-0159 18502888

[44] Zhang W, Hawse J, Huang Q, Sheets N, Miller KM, Horwitz J, Kantorow M. Decreased expression of ribosomal proteins in human age-related cataract. Invest Ophthalmol Vis Sci. 2002; 43:198–204. 11773032 PMC2831404

[45] Webster GC, Webster SL. Decline in synthesis of elongation factor one (EF-1) precedes the decreased synthesis of total protein in aging Drosophila melanogaster. Mech Ageing Dev. 1983; 22:121–28. 10.1016/0047-6374(83)90105-7 6415351

[46] Shikama N, Brack C. Changes in the expression of genes involved in protein synthesis during Drosophila aging. Gerontology. 1996; 42:123–36. 10.1159/000213783 8796371

[47] Muñoz MF, Argüelles S, Cano M, Marotta F, Ayala A. Aging and Oxidative Stress Decrease Pineal Elongation Factor 2: In Vivo Protective Effect of Melatonin in Young Rats Treated With Cumene Hydroperoxide. J Cell Biochem. 2017; 118:182–90. 10.1002/jcb.25624 27292877

[48] Kirstein-Miles J, Scior A, Deuerling E, Morimoto RI. The nascent polypeptide-associated complex is a key regulator of proteostasis. EMBO J. 2013; 32:1451–68. 10.1038/emboj.2013.87 23604074 PMC3655472

[49] Steffen KK, Dillin A. A Ribosomal Perspective on Proteostasis and Aging. Cell Metab. 2016; 23:1004–12. 10.1016/j.cmet.2016.05.013 27304502

[50] Dollé ME, Snyder WK, Gossen JA, Lohman PH, Vijg J. Distinct spectra of somatic mutations accumulated with age in mouse heart and small intestine. Proc Natl Acad Sci USA. 2000; 97:8403–08. 10.1073/pnas.97.15.8403 10900004 PMC26960

[51] Dollé ME, Snyder WK, Dunson DB, Vijg J. Mutational fingerprints of aging. Nucleic Acids Res. 2002; 30:545–49. 10.1093/nar/30.2.545 11788717 PMC99828

[52] Dollé ME, Giese H, Hopkins CL, Martus HJ, Hausdorff JM, Vijg J. Rapid accumulation of genome rearrangements in liver but not in brain of old mice. Nat Genet. 1997; 17:431–34. 10.1038/ng1297-431 9398844

[53] Guintivano J, Aryee MJ, Kaminsky ZA. A cell epigenotype specific model for the correction of brain cellular heterogeneity bias and its application to age, brain region and major depression. Epigenetics. 2013; 8:290–302. 10.4161/epi.23924 23426267 PMC3669121

[54] Horvath S, Mah V, Lu AT, Woo JS, Choi OW, Jasinska AJ, Riancho JA, Tung S, Coles NS, Braun J, Vinters HV, Coles LS. The cerebellum ages slowly according to the epigenetic clock. Aging (Albany NY). 2015; 7:294–306. 10.18632/aging.100742 26000617 PMC4468311

[55] Podolskiy DI, Lobanov AV, Kryukov GV, Gladyshev VN. Analysis of cancer genomes reveals basic features of human aging and its role in cancer development. Nat Commun. 2016; 7:12157. 10.1038/ncomms12157 27515585 PMC4990632

[56] Sehl ME, Henry JE, Storniolo AM, Ganz PA, Horvath S. DNA methylation age is elevated in breast tissue of healthy women. Breast Cancer Res Treat. 2017; 164:209–19. 10.1007/s10549-017-4218-4 28364215 PMC5487725

[57] Cook-Wiens E, Grotewiel MS. Dissociation between functional senescence and oxidative stress resistance in Drosophila. Exp Gerontol. 2002; 37:1347–57. 10.1016/S0531-5565(02)00096-7 12559404

[58] Mobley CB, Mumford PW, Kephart WC, Haun CT, Holland AM, Beck DT, Martin JS, Young KC, Anderson RG, Patel RK, Langston GL, Lowery RP, Wilson JM, Roberts MD. Aging in rats differentially affects markers of transcriptional and translational capacity in soleus and plantaris muscle. Front Physiol. 2017; 8:518. 10.3389/fphys.2017.00518 28775694 PMC5517446

[59] Ori A, Toyama BH, Harris MS, Bock T, Iskar M, Bork P, Ingolia NT, Hetzer MW, Beck M. Integrated Transcriptome and Proteome Analyses Reveal Organ-Specific Proteome Deterioration in Old Rats. Cell Syst. 2015; 1:224–37. 10.1016/j.cels.2015.08.012 27135913 PMC4802414

[60] Janssens GE, Meinema AC, González J, Wolters JC, Schmidt A, Guryev V, Bischoff R, Wit EC, Veenhoff LM, Heinemann M. Protein biogenesis machinery is a driver of replicative aging in yeast. eLife. 2015; 4:e08527. 10.7554/eLife.08527 26422514 PMC4718733

[61] Hu Z, Xia B, Postnikoff SD, Shen ZJ, Tomoiaga AS, Harkness TA, Seol JH, Li W, Chen K, Tyler JK. Ssd1 and Gcn2 suppress global translation efficiency in replicatively aged yeast while their activation extends lifespan. eLife. 2018; 7:7. 10.7554/eLife.35551 30117416 PMC6097839

[62] Malinovskaya EM, Ershova ES, Golimbet VE, Porokhovnik LN, Lyapunova NA, Kutsev SI, Veiko NN, Kostyuk SV. Copy Number of Human Ribosomal Genes With Aging: Unchanged Mean, but Narrowed Range and Decreased Variance in Elderly Group. Front Genet. 2018; 9:306. 10.3389/fgene.2018.00306 30131826 PMC6090032

[63] Janssens GE, Veenhoff LM. The Natural Variation in Lifespans of Single Yeast Cells Is Related to Variation in Cell Size, Ribosomal Protein, and Division Time. Csikász-Nagy A, editor. PLoS One. 2016; 11:e0167394. 10.1371/journal.pone.0167394 27907085 PMC5132237

[64] Mori N, Hiruta K, Funatsu Y, Goto S. Codon recognition fidelity of ribosomes at the first and second positions does not decrease during aging. Mech Ageing Dev. 1983; 22:1–10. 10.1016/0047-6374(83)90002-7 6621110

[65] Filion AM, Laughrea M. Translation fidelity in the aging mammal: studies with an accurate in vitro system on aged rats. Mech Ageing Dev. 1985; 29:125–42. 10.1016/0047-6374(85)90013-2 3974306

[66] Butzow JJ, McCool MG, Eichhorn GL. Does the capacity of ribosomes to control translation fidelity change with age? Mech Ageing Dev. 1981; 15:203–16. 10.1016/0047-6374(81)90075-0 7230911

[67] Luce MC, Bunn CL. Decreased accuracy of protein synthesis in extracts from aging human diploid fibroblasts. Exp Gerontol. 1989; 24:113–25. 10.1016/0531-5565(89)90022-3 2721600

[68] Wojtyk RI, Goldstein S. Fidelity of protein synthesis does not decline during aging of cultured human fibroblasts. J Cell Physiol. 1980; 103:299–303. 10.1002/jcp.1041030215 7440637

[69] Rattan SI. Synthesis, modification and turnover of proteins during aging. Adv Exp Med Biol. 2010; 694:1–13. 10.1007/978-1-4419-7002-2_1 20886752

[70] Stansfield I, Jones KM, Herbert P, Lewendon A, Shaw WV, Tuite MF. Missense translation errors in Saccharomyces cerevisiae. J Mol Biol. 1998; 282:13–24. 10.1006/jmbi.1998.1976 9733638

[71] Luce MC, Tschanz KD, Gotto DA, Bunn CL. The accuracy of protein synthesis in reticulocyte and HeLa cell lysates. Biochim Biophys Acta. 1985; 825:280–88. 10.1016/0167-4781(85)90015-6 4016118

[72] Loftfield RB. The frequency of errors in protein biosynthesis. Biochem J. 1963; 89:82–92. 10.1042/bj0890082 14097371 PMC1202275

[73] von der Haar T, Leadsham JE, Sauvadet A, Tarrant D, Adam IS, Saromi K, Laun P, Rinnerthaler M, Breitenbach-Koller H, Breitenbach M, Tuite MF, Gourlay CW. The control of translational accuracy is a determinant of healthy ageing in yeast. Open Biol. 2017; 7:160291. 10.1098/rsob.160291 28100667 PMC5303280

[74] Sudmant PH, Lee H, Dominguez D, Heiman M, Burge CB. Widespread Accumulation of Ribosome-Associated Isolated 3′ UTRs in Neuronal Cell Populations of the Aging Brain. Cell Reports. 2018; 25:2447–2456.e4. 10.1016/j.celrep.2018.10.094 30485811 PMC6354779

[75] Azpurua J, Ke Z, Chen IX, Zhang Q, Ermolenko DN, Zhang ZD, Gorbunova V, Seluanov A. Naked mole-rat has increased translational fidelity compared with the mouse, as well as a unique 28S ribosomal RNA cleavage. Proc Natl Acad Sci USA. 2013; 110:17350–55. 10.1073/pnas.1313473110 24082110 PMC3808608

[76] Ke Z, Mallik P, Johnson AB, Luna F, Nevo E, Zhang ZD, Gladyshev VN, Seluanov A, Gorbunova V. Translation fidelity coevolves with longevity. Aging Cell. 2017; 16:988–93. 10.1111/acel.12628 28707419 PMC5595694

[77] Bacher JM, Schimmel P. An editing-defective aminoacyl-tRNA synthetase is mutagenic in aging bacteria via the SOS response. Proc Natl Acad Sci USA. 2007; 104:1907–12. 10.1073/pnas.0610835104 17264207 PMC1794292

[78] Silar P, Picard M. Increased longevity of EF-1 alpha high-fidelity mutants in Podospora anserina. J Mol Biol. 1994; 235:231–36. 10.1016/S0022-2836(05)80029-4 8289244

[79] Schosserer M, Minois N, Angerer TB, Amring M, Dellago H, Harreither E, Calle-Perez A, Pircher A, Gerstl MP, Pfeifenberger S, Brandl C, Sonntagbauer M, Kriegner A, et al. Methylation of ribosomal RNA by NSUN5 is a conserved mechanism modulating organismal lifespan. Nat Commun. 2015; 6:6158. 10.1038/ncomms7158 25635753 PMC4317494

[80] Tavernarakis N. Ageing and the regulation of protein synthesis: a balancing act? Trends Cell Biol. 2008; 18:228–35. 10.1016/j.tcb.2008.02.004 18346894

[81] Kennedy BK, Kaeberlein M. Hot topics in aging research: protein translation, 2009. Aging Cell. 2009; 8:617–23. 10.1111/j.1474-9726.2009.00522.x 19747234 PMC3673879

[82] Syntichaki P, Troulinaki K, Tavernarakis N. eIF4E function in somatic cells modulates ageing in Caenorhabditis elegans. Nature. 2007; 445:922–26. 10.1038/nature05603 17277769

[83] Pan KZ, Palter JE, Rogers AN, Olsen A, Chen D, Lithgow GJ, Kapahi P. Inhibition of mRNA translation extends lifespan in Caenorhabditis elegans. Aging Cell. 2007; 6:111–19. 10.1111/j.1474-9726.2006.00266.x 17266680 PMC2745345

[84] Rogers AN, Chen D, McColl G, Czerwieniec G, Felkey K, Gibson BW, Hubbard A, Melov S, Lithgow GJ, Kapahi P. Life span extension via eIF4G inhibition is mediated by posttranscriptional remodeling of stress response gene expression in C. elegans. Cell Metab. 2011; 14:55–66. 10.1016/j.cmet.2011.05.010 21723504 PMC3220185

[85] Cattie DJ, Richardson CE, Reddy KC, Ness-Cohn EM, Droste R, Thompson MK, Gilbert WV, Kim DH. Mutations in Nonessential eIF3k and eIF3l Genes Confer Lifespan Extension and Enhanced Resistance to ER Stress in Caenorhabditis elegans. PLoS Genet. 2016; 12:e1006326. 10.1371/journal.pgen.1006326 27690135 PMC5045169

[86] Tohyama D, Yamaguchi A, Yamashita T. Inhibition of a eukaryotic initiation factor (eIF2Bdelta/F11A3.2) during adulthood extends lifespan in Caenorhabditis elegans. FASEB J. 2008; 22:4327–37. 10.1096/fj.08-112953 18728216

[87] Chen D, Pan KZ, Palter JE, Kapahi P. Longevity determined by developmental arrest genes in Caenorhabditis elegans. Aging Cell. 2007; 6:525–33. 10.1111/j.1474-9726.2007.00305.x 17521386 PMC2746107

[88] Curran SP, Ruvkun G. Lifespan regulation by evolutionarily conserved genes essential for viability. PLoS Genet. 2007; 3:e56. 10.1371/journal.pgen.0030056 17411345 PMC1847696

[89] Hamilton B, Dong Y, Shindo M, Liu W, Odell I, Ruvkun G, Lee SS. A systematic RNAi screen for longevity genes in C. elegans. Genes Dev. 2005; 19:1544–55. 10.1101/gad.1308205 15998808 PMC1172061

[90] Hansen M, Taubert S, Crawford D, Libina N, Lee SJ, Kenyon C. Lifespan extension by conditions that inhibit translation in Caenorhabditis elegans. Aging Cell. 2007; 6:95–110. 10.1111/j.1474-9726.2006.00267.x 17266679

[91] Henderson ST, Bonafè M, Johnson TE. daf-16 protects the nematode Caenorhabditis elegans during food deprivation. J Gerontol A Biol Sci Med Sci. 2006; 61:444–60. 10.1093/gerona/61.5.444 16720740

[92] Demontis F, Perrimon N. FOXO/4E-BP signaling in Drosophila muscles regulates organism-wide proteostasis during aging. Cell. 2010; 143:813–25. 10.1016/j.cell.2010.10.007 21111239 PMC3066043

[93] Teleman AA, Chen YW, Cohen SM. 4E-BP functions as a metabolic brake used under stress conditions but not during normal growth. Genes Dev. 2005; 19:1844–48. 10.1101/gad.341505 16103212 PMC1186183

[94] Kaeberlein M, Powers RW 3rd, Steffen KK, Westman EA, Hu D, Dang N, Kerr EO, Kirkland KT, Fields S, Kennedy BK. Regulation of yeast replicative life span by TOR and Sch9 in response to nutrients. Science. 2005; 310:1193–96. 10.1126/science.1115535 16293764

[95] Steffen KK, MacKay VL, Kerr EO, Tsuchiya M, Hu D, Fox LA, Dang N, Johnston ED, Oakes JA, Tchao BN, Pak DN, Fields S, Kennedy BK, Kaeberlein M. Yeast life span extension by depletion of 60s ribosomal subunits is mediated by Gcn4. Cell. 2008; 133:292–302. 10.1016/j.cell.2008.02.037 18423200 PMC2749658

[96] Smith ED, Tsuchiya M, Fox LA, Dang N, Hu D, Kerr EO, Johnston ED, Tchao BN, Pak DN, Welton KL, Promislow DE, Thomas JH, Kaeberlein M, Kennedy BK. Quantitative evidence for conserved longevity pathways between divergent eukaryotic species. Genome Res. 2008; 18:564–70. 10.1101/gr.074724.107 18340043 PMC2279244

[97] Belcour L, Begel O, Picard M. A site-specific deletion in mitochondrial DNA of Podospora is under the control of nuclear genes. Proc Natl Acad Sci USA. 1991; 88:3579–83. 10.1073/pnas.88.9.3579 2023905 PMC51495

[98] Stearns SC, Kaiser M. The effects of enhanced expression of elongation factor EF-1 alpha on lifespan in Drosophila melanogaster. IV. A summary of three experiments. Genetica. 1993; 91:167–82. 10.1007/BF01435996 8125267

[99] Shikama N, Ackermann R, Brack C. Protein synthesis elongation factor EF-1 alpha expression and longevity in Drosophila melanogaster. Proc Natl Acad Sci USA. 1994; 91:4199–203. 10.1073/pnas.91.10.4199 8183891 PMC43752

[100] Leprivier G, Remke M, Rotblat B, Dubuc A, Mateo AR, Kool M, Agnihotri S, El-Naggar A, Yu B, Somasekharan SP, Faubert B, Bridon G, Tognon CE, et al. The eEF2 kinase confers resistance to nutrient deprivation by blocking translation elongation. Cell. 2013; 153:1064–79. 10.1016/j.cell.2013.04.055 23706743 PMC4395874

[101] Hipkiss AR. On why decreasing protein synthesis can increase lifespan. Mech Ageing Dev. 2007; 128:412–14. 10.1016/j.mad.2007.03.002 17452047

[102] Solis GM, Kardakaris R, Valentine ER, Bar-Peled L, Chen AL, Blewett MM, McCormick MA, Williamson JR, Kennedy B, Cravatt BF, Petrascheck M. Translation attenuation by minocycline enhances longevity and proteostasis in old post-stress-responsive organisms. eLife. 2018; 7:7. 10.7554/eLife.40314 30479271 PMC6257811

[103] Wieser W, Krumschnabel G. Hierarchies of ATP-consuming processes: direct compared with indirect measurements, and comparative aspects. Biochem J. 2001; 355:389–95. 10.1042/bj3550389 11284726 PMC1221750

[104] Mathis AD, Naylor BC, Carson RH, Evans E, Harwell J, Knecht J, Hexem E, Peelor FF 3rd, Miller BF, Hamilton KL, Transtrum MK, Bikman BT, Price JC. Mechanisms of In Vivo Ribosome Maintenance Change in Response to Nutrient Signals. Mol Cell Proteomics. 2017; 16:243–54. 10.1074/mcp.M116.063255 27932527 PMC5294211

[105] Pestov DG, Shcherbik N. Rapid cytoplasmic turnover of yeast ribosomes in response to rapamycin inhibition of TOR. Mol Cell Biol. 2012; 32:2135–44. 10.1128/MCB.06763-11 22451491 PMC3372233

[106] Holbrook MA, Menninger JR. Erythromycin slows aging of Saccharomyces cerevisiae. J Gerontol A Biol Sci Med Sci. 2002; 57:B29–36. 10.1093/gerona/57.1.B29 11773204

[107] Heeren G, Rinnerthaler M, Laun P, von Seyerl P, Kössler S, Klinger H, Hager M, Bogengruber E, Jarolim S, Simon-Nobbe B, Schüller C, Carmona-Gutierrez D, Breitenbach-Koller L, et al. The mitochondrial ribosomal protein of the large subunit, Afo1p, determines cellular longevity through mitochondrial back-signaling via TOR1. Aging (Albany NY). 2009; 1:622–36. 10.18632/aging.100065 20157544 PMC2806038

[108] Delaney JR, Ahmed U, Chou A, Sim S, Carr D, Murakami CJ, Schleit J, Sutphin GL, An EH, Castanza A, Fletcher M, Higgins S, Jelic M, et al. Stress profiling of longevity mutants identifies Afg3 as a mitochondrial determinant of cytoplasmic mRNA translation and aging. Aging Cell. 2013; 12:156–66. 10.1111/acel.12032 23167605 PMC3687586

[109] Postnikoff SD, Johnson JE, Tyler JK. The integrated stress response in budding yeast lifespan extension. Microb Cell. 2017; 4:368–75. 10.15698/mic2017.11.597 29167799 PMC5695854

[110] Sherman MY, Qian SB, Powers ET, Balch WE, Ravikumar B, Rubinsztein DC, Rubinsztein DC, et al. Less is more: improving proteostasis by translation slow down. Trends Biochem Sci. 2013; 38:585–91. 10.1016/j.tibs.2013.09.003 24126073

[111] Proud CG. Phosphorylation and Signal Transduction Pathways in Translational Control. Cold Spring Harb Perspect Biol. 2018; Epub ahead of print. 10.1101/cshperspect.a033050 29959191 PMC6601458

[112] Powers RW 3rd, Kaeberlein M, Caldwell SD, Kennedy BK, Fields S. Extension of chronological life span in yeast by decreased TOR pathway signaling. Genes Dev. 2006; 20:174–84. 10.1101/gad.1381406 16418483 PMC1356109

[113] Bjedov I, Toivonen JM, Kerr F, Slack C, Jacobson J, Foley A, Partridge L. Mechanisms of life span extension by rapamycin in the fruit fly Drosophila melanogaster. Cell Metab. 2010; 11:35–46. 10.1016/j.cmet.2009.11.010 20074526 PMC2824086

[114] Miller RA, Harrison DE, Astle CM, Baur JA, Boyd AR, de Cabo R, Fernandez E, Flurkey K, Javors MA, Nelson JF, Orihuela CJ, Pletcher S, Sharp ZD, et al. Rapamycin, but not resveratrol or simvastatin, extends life span of genetically heterogeneous mice. J Gerontol A Biol Sci Med Sci. 2011; 66:191–201. 10.1093/gerona/glq178 20974732 PMC3021372

[115] Robida-Stubbs S, Glover-Cutter K, Lamming DW, Mizunuma M, Narasimhan SD, Neumann-Haefelin E, Sabatini DM, Blackwell TK. TOR signaling and rapamycin influence longevity by regulating SKN-1/Nrf and DAF-16/FoxO. Cell Metab. 2012; 15:713–24. 10.1016/j.cmet.2012.04.007 22560223 PMC3348514

[116] Blagosklonny MV. From rapalogs to anti-aging formula. Oncotarget. 2017; 8:35492–507. 10.18632/oncotarget.18033 28548953 PMC5482593

[117] Lamming DW, Ye L, Sabatini DM, Baur JA. Rapalogs and mTOR inhibitors as anti-aging therapeutics. J Clin Invest. 2013; 123:980–89. 10.1172/JCI64099 23454761 PMC3582126

[118] Eltschinger S, Loewith R. TOR Complexes and the Maintenance of Cellular Homeostasis. Trends Cell Biol. 2016; 26:148–59. 10.1016/j.tcb.2015.10.003 26546292

[119] Thoreen CC. The molecular basis of mTORC1-regulated translation. Biochem Soc Trans. 2017; 45:213–21. 10.1042/BST20160072 28202675

[120] Meyuhas O, Kahan T. The race to decipher the top secrets of TOP mRNAs. Biochim Biophys Acta. 2015; 1849:801–11. 10.1016/j.bbagrm.2014.08.015 25234618

[121] Ben-Sahra I, Manning BD. mTORC1 signaling and the metabolic control of cell growth. Curr Opin Cell Biol. 2017; 45:72–82. 10.1016/j.ceb.2017.02.012 28411448 PMC5545101

[122] Nandagopal N, Roux PP. Regulation of global and specific mRNA translation by the mTOR signaling pathway. Translation (Austin). 2015; 3:e983402. 10.4161/21690731.2014.983402 26779414 PMC4682803

[123] Huo Y, Iadevaia V, Yao Z, Kelly I, Cosulich S, Guichard S, Foster LJ, Proud CG. Stable isotope-labelling analysis of the impact of inhibition of the mammalian target of rapamycin on protein synthesis. Biochem J. 2012; 444:141–51. 10.1042/BJ20112107 22428559

[124] Thoreen CC, Kang SA, Chang JW, Liu Q, Zhang J, Gao Y, Reichling LJ, Sim T, Sabatini DM, Gray NS. An ATP-competitive mammalian target of rapamycin inhibitor reveals rapamycin-resistant functions of mTORC1. J Biol Chem. 2009; 284:8023–32. 10.1074/jbc.M900301200 19150980 PMC2658096

[125] Hsieh AC, Liu Y, Edlind MP, Ingolia NT, Janes MR, Sher A, Shi EY, Stumpf CR, Christensen C, Bonham MJ, Wang S, Ren P, Martin M, et al. The translational landscape of mTOR signalling steers cancer initiation and metastasis. Nature. 2012; 485:55–61. 10.1038/nature10912 22367541 PMC3663483

[126] Choo AY, Yoon SO, Kim SG, Roux PP, Blenis J. Rapamycin differentially inhibits S6Ks and 4E-BP1 to mediate cell-type-specific repression of mRNA translation. Proc Natl Acad Sci USA. 2008; 105:17414–19. 10.1073/pnas.0809136105 18955708 PMC2582304

[127] Batool A, Aashaq S, Andrabi KI. Reappraisal to the study of 4E-BP1 as an mTOR substrate - A normative critique. Eur J Cell Biol. 2017; 96:325–36. 10.1016/j.ejcb.2017.03.013 28427795

[128] Shahbazian D, Roux PP, Mieulet V, Cohen MS, Raught B, Taunton J, Hershey JW, Blenis J, Pende M, Sonenberg N. The mTOR/PI3K and MAPK pathways converge on eIF4B to control its phosphorylation and activity. EMBO J. 2006; 25:2781–91. 10.1038/sj.emboj.7601166 16763566 PMC1500846

[129] Kang SA, Pacold ME, Cervantes CL, Lim D, Lou HJ, Ottina K, Gray NS, Turk BE, Yaffe MB, Sabatini DM. mTORC1 phosphorylation sites encode their sensitivity to starvation and rapamycin. Science. 2013; 341:1236566. 10.1126/science.1236566 23888043 PMC3771538

[130] Shagam LI, Terenin IM, Andreev DE, Dunaevsky JE, Dmitriev SE. In vitro activity of human translation initiation factor eIF4B is not affected by phosphomimetic amino acid substitutions S422D and S422E. Biochimie. 2012; 94:2484–90. 10.1016/j.biochi.2012.06.021 22750809

[131] Bahrami-B F, Ataie-Kachoie P, Pourgholami MH, Morris DL. p70 Ribosomal protein S6 kinase (Rps6kb1): an update. J Clin Pathol. 2014; 67:1019–25. 10.1136/jclinpath-2014-202560 25100792

[132] Johnson SC, Rabinovitch PS, Kaeberlein M. mTOR is a key modulator of ageing and age-related disease. Nature. 2013; 493:338–45. 10.1038/nature11861 23325216 PMC3687363

[133] Jia K, Chen D, Riddle DL. The TOR pathway interacts with the insulin signaling pathway to regulate C. elegans larval development, metabolism and life span. Development. 2004; 131:3897–906. 10.1242/dev.01255 15253933

[134] Kapahi P, Zid BM, Harper T, Koslover D, Sapin V, Benzer S. Regulation of lifespan in Drosophila by modulation of genes in the TOR signaling pathway. Curr Biol. 2004; 14:885–90. 10.1016/j.cub.2004.03.059 15186745 PMC2754830

[135] Lamming DW, Ye L, Katajisto P, Goncalves MD, Saitoh M, Stevens DM, Davis JG, Salmon AB, Richardson A, Ahima RS, Guertin DA, Sabatini DM, Baur JA. Rapamycin-induced insulin resistance is mediated by mTORC2 loss and uncoupled from longevity. Science. 2012; 335:1638–43. 10.1126/science.1215135 22461615 PMC3324089

[136] Baar EL, Carbajal KA, Ong IM, Lamming DW. Sex- and tissue-specific changes in mTOR signaling with age in C57BL/6J mice. Aging Cell. 2016; 15:155–66. 10.1111/acel.12425 26695882 PMC4717274

[137] Zid BM, Rogers AN, Katewa SD, Vargas MA, Kolipinski MC, Lu TA, Benzer S, Kapahi P. 4E-BP extends lifespan upon dietary restriction by enhancing mitochondrial activity in Drosophila. Cell. 2009; 139:149–60. 10.1016/j.cell.2009.07.034 19804760 PMC2759400

[138] Selman C, Tullet JM, Wieser D, Irvine E, Lingard SJ, Choudhury AI, Claret M, Al-Qassab H, Carmignac D, Ramadani F, Woods A, Robinson IC, Schuster E, et al. Ribosomal protein S6 kinase 1 signaling regulates mammalian life span. Science. 2009; 326:140–44. 10.1126/science.1177221 19797661 PMC4954603

[139] Andreev DE, Dmitriev SE, Loughran G, Terenin IM, Baranov PV, Shatsky IN. Translation control of mRNAs encoding mammalian translation initiation factors. Gene. 2018; 651:174–82. 10.1016/j.gene.2018.02.013 29414693

[140] Thoreen CC, Chantranupong L, Keys HR, Wang T, Gray NS, Sabatini DM. A unifying model for mTORC1-mediated regulation of mRNA translation. Nature. 2012; 485:109–13. 10.1038/nature11083 22552098 PMC3347774

[141] Shatsky IN, Dmitriev SE, Andreev DE, Terenin IM. Transcriptome-wide studies uncover the diversity of modes of mRNA recruitment to eukaryotic ribosomes. Crit Rev Biochem Mol Biol. 2014; 49:164–77. 10.3109/10409238.2014.887051 24520918

[142] Damgaard CK, Lykke-Andersen J. Translational coregulation of 5'TOP mRNAs by TIA-1 and TIAR. Genes Dev. 2011; 25:2057–68. 10.1101/gad.17355911 21979918 PMC3197204

[143] Miloslavski R, Cohen E, Avraham A, Iluz Y, Hayouka Z, Kasir J, Mudhasani R, Jones SN, Cybulski N, Rüegg MA, Larsson O, Gandin V, Rajakumar A, et al. Oxygen sufficiency controls TOP mRNA translation via the TSC-Rheb-mTOR pathway in a 4E-BP-independent manner. J Mol Cell Biol. 2014; 6:255–66. 10.1093/jmcb/mju008 24627160 PMC4034726

[144] Tcherkezian J, Cargnello M, Romeo Y, Huttlin EL, Lavoie G, Gygi SP, Roux PP. Proteomic analysis of cap-dependent translation identifies LARP1 as a key regulator of 5'TOP mRNA translation. Genes Dev. 2014; 28:357–71. 10.1101/gad.231407.113 24532714 PMC3937514

[145] Lahr RM, Fonseca BD, Ciotti GE, Al-Ashtal HA, Jia JJ, Niklaus MR, Blagden SP, Alain T, Berman AJ. La-related protein 1 (LARP1) binds the mRNA cap, blocking eIF4F assembly on TOP mRNAs. eLife. 2017; 6:e24146. 10.7554/eLife.24146 28379136 PMC5419741

[146] Fonseca BD, Zakaria C, Jia JJ, Graber TE, Svitkin Y, Tahmasebi S, Healy D, Hoang HD, Jensen JM, Diao IT, Lussier A, Dajadian C, Padmanabhan N, et al. La-related Protein 1 (LARP1) Represses Terminal Oligopyrimidine (TOP) mRNA Translation Downstream of mTOR Complex 1 (mTORC1). J Biol Chem. 2015; 290:15996–6020. 10.1074/jbc.M114.621730 25940091 PMC4481205

[147] Philippe L, Vasseur JJ, Debart F, Thoreen CC. La-related protein 1 (LARP1) repression of TOP mRNA translation is mediated through its cap-binding domain and controlled by an adjacent regulatory region. Nucleic Acids Res. 2018; 46:1457–69. 10.1093/nar/gkx1237 29244122 PMC5814973

[148] Hong S, Freeberg MA, Han T, Kamath A, Yao Y, Fukuda T, Suzuki T, Kim JK, Inoki K. LARP1 functions as a molecular switch for mTORC1-mediated translation of an essential class of mRNAs. eLife. 2017; 6:6. 10.7554/eLife.25237 28650797 PMC5484620

[149] Tamarkin-Ben-Harush A, Vasseur JJ, Debart F, Ulitsky I, Dikstein R. Cap-proximal nucleotides via differential eIF4E binding and alternative promoter usage mediate translational response to energy stress. eLife. 2017; 6:6. 10.7554/eLife.21907 28177284 PMC5308895

[150] Tebaldi T, Zuccotti P, Peroni D, Köhn M, Gasperini L, Potrich V, Bonazza V, Dudnakova T, Rossi A, Sanguinetti G, Conti L, Macchi P, D’Agostino V, et al. HuD Is a Neural Translation Enhancer Acting on mTORC1-Responsive Genes and Counteracted by the Y3 Small Non-coding RNA. Mol Cell. 2018; 71:256–270.e10. 10.1016/j.molcel.2018.06.032 30029004 PMC6060611

[151] Chang JW, Zhang W, Yeh HS, de Jong EP, Jun S, Kim KH, Bae SS, Beckman K, Hwang TH, Kim KS, Kim DH, Griffin TJ, Kuang R, Yong J. mRNA 3′-UTR shortening is a molecular signature of mTORC1 activation. Nat Commun. 2015; 6:7218. 10.1038/ncomms8218 26074333

[152] Chen M, Lyu G, Han M, Nie H, Shen T, Chen W, Niu Y, Song Y, Li X, Li H, Chen X, Wang Z, Xia Z, et al. 3′ UTR lengthening as a novel mechanism in regulating cellular senescence. Genome Res. 2018; 28:285–94. 10.1101/gr.224451.117 29440281 PMC5848608

[153] Meyer KD, Patil DP, Zhou J, Zinoviev A, Skabkin MA, Elemento O, Pestova TV, Qian SB, Jaffrey SR. 5′ UTR m(6)A Promotes Cap-Independent Translation. Cell. 2015; 163:999–1010. 10.1016/j.cell.2015.10.012 26593424 PMC4695625

[154] Coots RA, Liu XM, Mao Y, Dong L, Zhou J, Wan J, Zhang X, Qian SB. m^6^A Facilitates eIF4F-Independent mRNA Translation. Mol Cell. 2017; 68:504–514.e7. 10.1016/j.molcel.2017.10.002 29107534 PMC5913006

[155] Li A, Chen YS, Ping XL, Yang X, Xiao W, Yang Y, Sun HY, Zhu Q, Baidya P, Wang X, Bhattarai DP, Zhao YL, Sun BF, Yang YG. Cytoplasmic m^6^A reader YTHDF3 promotes mRNA translation. Cell Res. 2017; 27:444–47. 10.1038/cr.2017.10 28106076 PMC5339832

[156] Zhou J, Wan J, Gao X, Zhang X, Jaffrey SR, Qian SB. Dynamic m(6)A mRNA methylation directs translational control of heat shock response. Nature. 2015; 526:591–94. 10.1038/nature15377 26458103 PMC4851248

[157] Sinvani H, Haimov O, Svitkin Y, Sonenberg N, Tamarkin-Ben-Harush A, Viollet B, Dikstein R. Translational tolerance of mitochondrial genes to metabolic energy stress involves TISU and eIF1-eIF4GI cooperation in start codon selection. Cell Metab. 2015; 21:479–92. 10.1016/j.cmet.2015.02.010 25738462

[158] Lee AS, Kranzusch PJ, Doudna JA, Cate JH. eIF3d is an mRNA cap-binding protein that is required for specialized translation initiation. Nature. 2016; 536:96–99. 10.1038/nature18954 27462815 PMC5003174

[159] Ryu I, Kim YK. Translation initiation mediated by nuclear cap-binding protein complex. BMB Rep. 2017; 50:186–93. 10.5483/BMBRep.2017.50.4.007 28088948 PMC5437962

[160] Chen HH, Yu HI, Yang MH, Tarn WY. DDX3 Activates CBC-eIF3-Mediated Translation of uORF-Containing Oncogenic mRNAs to Promote Metastasis in HNSCC. Cancer Res. 2018; 78:4512–23. 10.1158/0008-5472.CAN-18-0282 29921696

[161] Ho JJ, Lee S. A Cap for Every Occasion: Alternative eIF4F Complexes. Trends Biochem Sci. 2016; 41:821–23. 10.1016/j.tibs.2016.05.009 27283511 PMC5045779

[162] Bush MS, Hutchins AP, Jones AM, Naldrett MJ, Jarmolowski A, Lloyd CW, Doonan JH. Selective recruitment of proteins to 5′ cap complexes during the growth cycle in Arabidopsis. Plant J. 2009; 59:400–12. 10.1111/j.1365-313X.2009.03882.x 19453450

[163] Soto-Rifo R, Rubilar PS, Ohlmann T. The DEAD-box helicase DDX3 substitutes for the cap-binding protein eIF4E to promote compartmentalized translation initiation of the HIV-1 genomic RNA. Nucleic Acids Res. 2013; 41:6286–99. 10.1093/nar/gkt306 23630313 PMC3695493

[164] Min KW, Zealy RW, Davila S, Fomin M, Cummings JC, Makowsky D, Mcdowell CH, Thigpen H, Hafner M, Kwon SH, Georgescu C, Wren JD, Yoon JH. Profiling of m6A RNA modifications identified an age-associated regulation of AGO2 mRNA stability. Aging Cell. 2018; 17:e12753. 10.1111/acel.12753 29573145 PMC5946072

[165] Uno M, Nishida E. Lifespan-regulating genes in *C. elegans.* NPJ Aging Mech Dis. 2016; 2:16010. 10.1038/npjamd.2016.10 28721266 PMC5514992

[166] González A, Hall MN. Nutrient sensing and TOR signaling in yeast and mammals. EMBO J. 2017; 36:397–408. 10.15252/embj.201696010 28096180 PMC5694944

[167] Williamson D, Gallagher P, Harber M, Hollon C, Trappe S. Mitogen-activated protein kinase (MAPK) pathway activation: effects of age and acute exercise on human skeletal muscle. J Physiol. 2003; 547:977–87. 10.1113/jphysiol.2002.036673 12562918 PMC2342728

[168] Darnell AM, Subramaniam AR, O’Shea EK. Translational Control through Differential Ribosome Pausing during Amino Acid Limitation in Mammalian Cells. Mol Cell. 2018; 71:229–243.e11. 10.1016/j.molcel.2018.06.041 30029003 PMC6516488

[169] Hann SR, Sloan-Brown K, Spotts GD. Translational activation of the non-AUG-initiated c-myc 1 protein at high cell densities due to methionine deprivation. Genes Dev. 1992; 6:1229–40. 10.1101/gad.6.7.1229 1628829

[170] Mazor KM, Dong L, Mao Y, Swanda RV, Qian SB, Stipanuk MH. Effects of single amino acid deficiency on mRNA translation are markedly different for methionine versus leucine. Sci Rep. 2018; 8:8076. 10.1038/s41598-018-26254-2 29795412 PMC5967319

[171] Young SK, Wek RC. Upstream Open Reading Frames Differentially Regulate Gene-specific Translation in the Integrated Stress Response. J Biol Chem. 2016; 291:16927–35. 10.1074/jbc.R116.733899 27358398 PMC5016099

[172] Hinnebusch AG, Ivanov IP, Sonenberg N. Translational control by 5′-untranslated regions of eukaryotic mRNAs. Science. 2016; 352:1413–16. 10.1126/science.aad9868 27313038 PMC7422601

[173] Nikonorova IA, Mirek ET, Signore CC, Goudie MP, Wek RC, Anthony TG. Time-resolved analysis of amino acid stress identifies eIF2 phosphorylation as necessary to inhibit mTORC1 activity in liver. J Biol Chem. 2018; 293:5005–15. 10.1074/jbc.RA117.001625 29449374 PMC5892569

[174] Cherkasova VA, Hinnebusch AG. Translational control by TOR and TAP42 through dephosphorylation of eIF2α kinase GCN2. Genes Dev. 2003; 17:859–72. 10.1101/gad.1069003 12654728 PMC196024

[175] Pakos-Zebrucka K, Koryga I, Mnich K, Ljujic M, Samali A, Gorman AM. The integrated stress response. EMBO Rep. 2016; 17:1374–95. 10.15252/embr.201642195 27629041 PMC5048378

[176] Su KH, Dai C. mTORC1 senses stresses: coupling stress to proteostasis. BioEssays. 2017; 39:1600268. 10.1002/bies.201600268 28295473 PMC5557634

[177] Lenox AR, Bhootada Y, Gorbatyuk O, Fullard R, Gorbatyuk M. Unfolded protein response is activated in aged retinas. Neurosci Lett. 2015; 609:30–35. 10.1016/j.neulet.2015.10.019 26467812 PMC4679557

[178] Apfeld J, O’Connor G, McDonagh T, DiStefano PS, Curtis R. The AMP-activated protein kinase AAK-2 links energy levels and insulin-like signals to lifespan in C. elegans. Genes Dev. 2004; 18:3004–09. 10.1101/gad.1255404 15574588 PMC535911

[179] Haustead DJ, Stevenson A, Saxena V, Marriage F, Firth M, Silla R, Martin L, Adcroft KF, Rea S, Day PJ, Melton P, Wood FM, Fear MW. Transcriptome analysis of human ageing in male skin shows mid-life period of variability and central role of NF-κB. Sci Rep. 2016; 6:26846. 10.1038/srep26846 27229172 PMC4882522

[180] Roux PP, Shahbazian D, Vu H, Holz MK, Cohen MS, Taunton J, Sonenberg N, Blenis J. RAS/ERK signaling promotes site-specific ribosomal protein S6 phosphorylation via RSK and stimulates cap-dependent translation. J Biol Chem. 2007; 282:14056–64. 10.1074/jbc.M700906200 17360704 PMC3618456

